# Application of microdeletion and microduplication screening in preimplantation genetic testing: a case report

**DOI:** 10.1186/s13256-026-05832-3

**Published:** 2026-01-22

**Authors:** Maria Katz, Ben Podgursky, Shenglai Li, Qinnan Zhang, Daniel Shapiro, Monica Pasternak, Noor Siddiqui, Funda Suer, Yuntao Xia

**Affiliations:** 1 Orchid Health, Palo Alto, CA 94301 USA; 2 Orchid Health Genomic Lab, 4022 Stirrup Creek Dr, STE312, Durham, NC 27703 USA; 3 Reproductive Biology Associates, Atlanta, GA 30342 USA; 4 Spring Fertility, San Francisco, CA 94109 USA

**Keywords:** Preimplantation genetic testing–whole genome sequencing, Microdeletion, Microduplication

## Abstract

**Background:**

Microdeletions and microduplications are chromosomal variants ranging up to 3 Mb in size. These abnormalities often arise spontaneously and have significant clinical implications, including developmental delay and congenital anomalies. Given the resolution of conventional preimplantation genetic testing for aneuploidy is > 5–10 Mb, these clinically significant abnormalities are missed. This study presents two cases where preimplantation genetic testing–whole genome sequencing successfully identified small, clinically significant abnormalities.

**Case presentation:**

In the first case, a couple undergoing *in vitro* fertilization opted to pursue preimplantation genetic testing–whole genome sequencing. Both the female patient (of East Asian ancestry, 32 years old) and the male patient (of European ancestry, 31 years old) did not disclose any history of genetic conditions. A screen of 50 pathogenic microdeletion and microduplication regions was performed in trophectoderm biopsies, identifying a 1.7-Mb microduplication at Xp22.31, linked to seizures, across multiple samples. The female patient later revealed a history of seizures, previously unaware of a genetic cause, highlighting the clinical relevance of this finding. In the second case, a couple of South Asian ancestry (34-year-old female and 36-year-old male) opted to pursue preimplantation genetic testing–whole genome sequencing for targeted screening of a 412-kb duplication on chromosome 10. Since the duplication had originated de novo in the male patient, the patient lacked informative family members for traditional preimplantation genetic testing probe development and was rejected by other preimplantation genetic testing laboratories. However, preimplantation genetic testing–whole genome sequencing successfully accommodated this region, enabling the identification of unaffected embryos.

**Conclusion:**

Preimplantation genetic testing–whole genome sequencing offers higher chromosomal resolution than conventional preimplantation genetic testing, enabling general microdeletion/duplication screening as well as the detection of difficult chromosomal variations. Patients undergoing *in vitro* fertilization now have the option to screen for additional clinically relevant conditions and explore family planning solutions for familial complex chromosomal abnormalities.

**Supplementary Information:**

The online version contains supplementary material available at 10.1186/s13256-026-05832-3.

## Background

Microdeletions and microduplications (microdel/dups) are chromosomal abnormalities typically ranging from 1 to 3 megabases (Mb) in size [1]. These changes are often spontaneous and are present in about 5% of patients with unexplained intellectual disabilities [2–6]. While microdels/dups can occur throughout the genome, several recurrent regions are well documented. For example, 22q11.2 deletion syndrome, commonly referred to as DiGeorge syndrome, involves various breakpoints and deletion sizes within this region (1.5–2.5 Mb). These variations can also be associated with a range of clinical manifestations [7]. Other examples include the 7q11.23 deletion (~1.5 M), associated with Williams–Beuren Syndrome, and the 4p16.3 deletion (1.7 Mb), linked to Wolf–Hirschhorn syndrome. Numerous clinical databases and studies have cataloged recurrent microdels/dups and their associated symptoms, ranging from mild to severe [8].

Given the clinical relevance of microdel/dups, several are now included in non-invasive prenatal testing (NIPT), enabling detection as early as 8 weeks of gestation and providing patients with more comprehensive genetic insights [9]. However, parents rarely know that microdel/dups screening can be extended to preimplantation genetic testing (PGT) during *in vitro* fertilization (IVF).

Traditional preimplantation genetic testing platforms cannot achieve the necessary resolution of 1–3 Mb for identifying microdels/dups. However, preimplantation genetic testing with whole genome sequencing (PGT–WGS), a new type of PGT, allows for screening of known recurrent pathogenic microdel/dup regions (Supplementary Table S1) and can accommodate additional regions upon request, with a resolution limit of approximately 400 kilobases (kb) [10].

In this study, we present two cases using PGT–WGS (Supplementary Fig. S1). In the first case, general microdel/dup screening of a batch of embryo biopsies identified a previously unknown inherited microduplication syndrome associated with seizures. In the second case, PGT–WGS allowed for the screening of a known 400 kb microduplication syndrome that other PGT providers had rejected owing to its size and its de novo nature.

## Case presentation

### Case 1: identification of a pathogenic microduplication associated with seizures in a trophectoderm biopsy without parental DNA

A 30-year-old male (of European ancestry) and female (of East Asian ancestry) couple underwent IVF for fertility preservation and elected to perform PGT–WGS, including screening for aneuploidy (PGT-A) plus targeted microdel/dup analysis. Since the couple had no known genetic conditions and carrier screening revealed no X-linked or shared autosomal recessive conditions, no specific variants were requested for the test. The couple shared no significant medical history or pre-existing conditions with their IVF providers.

**Fig. 1:**
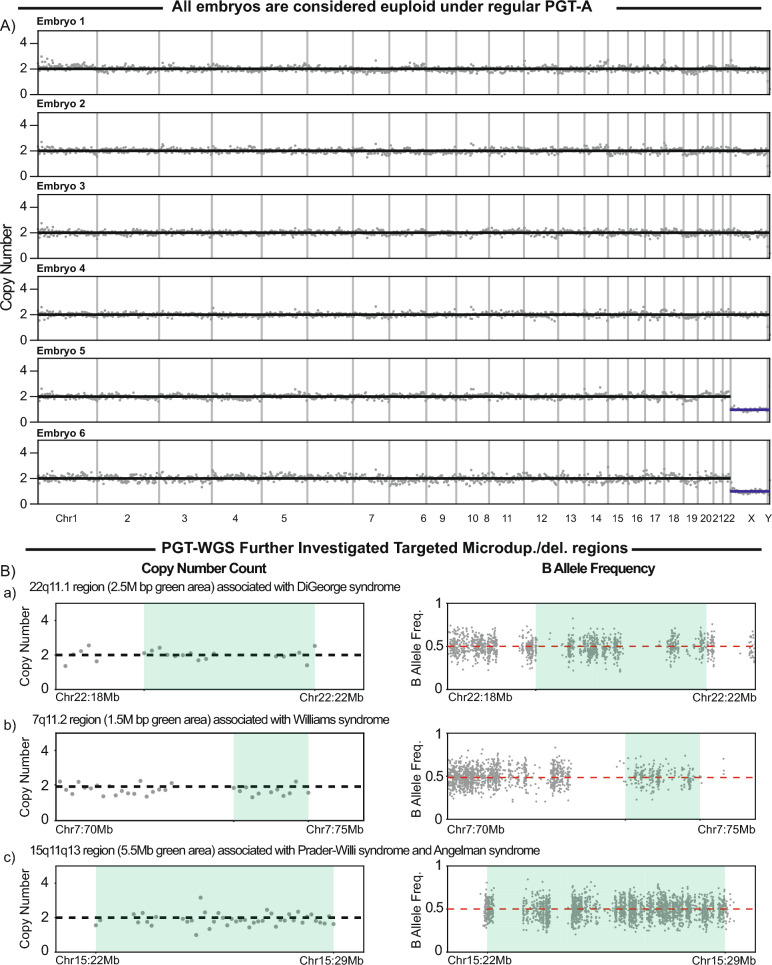
**A** Copy number analysis of representative embryos. **B** Copy number analysis and B-allele frequency analysis of embryo 5, highlighting regions representing microdeletions and microduplications

A total of 14 embryo biopsies underwent PGT–WGS beginning with PGT-A; 11 were euploid and underwent additional analysis. Further analysis revealed that six of these embryos had a 1.7-Mb microduplication at Xp22.31. Representative embryos are shown in Fig. 1.

With traditional PGT-A, as shown in the chromosome copy number pattern in Fig. 1A, embryos with a microduplication would be reported as euploid. This is owing to PGT-A’s detection limit, which typically identifies variations larger than 5–10 Mb, overlooking smaller structural variants such as microduplications.

PGT–WGS includes an additional analysis of 50 pathogenic microdels/dups. For each region of interest, chromosome count is used to evaluate the copy number, and then the result is confirmed by analyzing patterns of B-allele frequency (BAF). For an embryo that screens negative, with two copies of a chromosome, heterozygous variants will have a BAF of approximately 0.5 (Fig. 1B) [11].

**Fig. 2:**
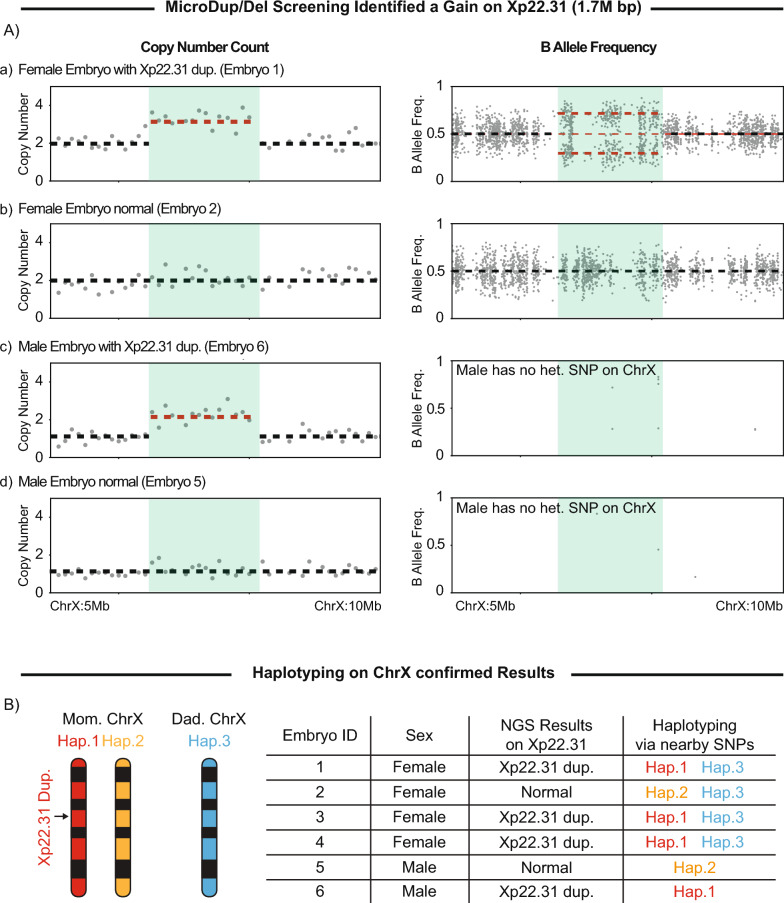
**A** Copy number analysis and B-allele frequency analysis of representing embryos, highlighting the Xp22.31 region. **B** The summary table presents the Xp22.31 results for each embryo. The haplotyping analysis confirmed the Xp22.1 findings accordingly

Using embryo 5 as an example, the 22q11.1 region displayed two chromosome copies, consistent with the observed BAF pattern, showing that the embryo is screened negative for DiGeorge syndrome (Fig. 1B-a). Similarly, the 7q11.2 region, linked to Williams–Beuren syndrome, also displayed two copies, supported by BAF, indicating no abnormalities (Fig. 1B-b). This can be compared with the Xp22.31 region, where a clear 1.7-Mb microduplication is observed across multiple embryos (Fig. 2A&B). In affected female embryos, the duplicated chromosomal segment was confirmed by a split pattern of BAF. Within the duplication, the BAF for heterozygous variants, in theory, would shift to either 0.33 or 0.66, matching precisely with the observed results (Fig. 2A-a). In male embryos, BAF analysis is not possible because of the lack of heterozygosity on the single X chromosome. However, the copy number count successfully identified the microduplication (Fig. 2A-c). Given that multiple male and female embryos were found to carry the duplication, the affected X chromosome was likely inherited from the female patient.

Haplotyping was then performed to confirm the PGT–WGS findings. In the absence of parental DNA, an affected male embryo served as the proband for this analysis. Haplotyping identifies specific allele combinations inherited together within a targeted genomic region. The results were consistent with PGT–WGS and aligned with the expected inheritance pattern from the female patient (Fig. 2B).

Duplications in the Xp22.31 region are associated with mild-to-moderate intellectual disability, developmental delays, and, in some cases, epilepsy or seizure activity [12]. In some individuals, the duplication may lead to focal seizures or generalized seizures that affect the entire brain, manifesting as absence seizures (brief lapses in awareness) or tonic–clonic seizures (stiffening and rhythmic jerking). As this microduplication is on the X chromosome, males are more likely to be symptomatic than females. However, females may also exhibit symptoms owing to skewed X-inactivation. During post-test genetic counseling, the patient disclosed a personal history of seizures and was advised to consult with a geneticist for confirmatory testing and further evaluation of symptoms.

The female patient then underwent follow-up testing, which confirmed that she carries the Xp22.31 variant, consistent with the PGT–WGS findings.

This information enabled the couple to decide whether to prioritize embryos without the Xp22.31 duplication for transfer. The American Society of Reproductive Medicine (ASRM) has extensively discussed the ethical considerations surrounding the transfer of embryos with late-onset or manageable conditions. They concluded that, with appropriate counseling, such transfers may be considered acceptable on the basis of individual circumstances and informed consent [13].

PGT–WGS allows for detecting microdeletions and microduplications as small as > 400 kb, compared with the threshold of traditional methods of > 5–10 Mb. A targeted approach is employed to screen for 50 well-established pathogenic microdeletion and microduplication regions to ensure that only clinically relevant results are reported.

### Case 2: targeted embryo screening for a paternal microduplication at 10q24.31q24.32

A couple of South Asian ancestry (34-year-old female and 36-year-old male) opted to pursue PGT–WGS. The male patient with split-hand/foot malformation, attributed to a previously identified 412-kb duplication at 10q24.31q24.32, sought to screen his future embryos. The duplication, which spans five genes (LBX1, FBXW4, BTRC, POLL, and DPCD), is inherited in an autosomal dominant manner, conferring a 50% chance of transmission to offspring, resulting in a similar phenotype.

According to the male patient, follow-up testing of his parents revealed they were negative for this duplication, indicating a de novo origin in the patient. Owing to the lack of informative family samples for probe development and the size of the duplication, traditional PGT laboratories declined the case.

**Fig. 3:**
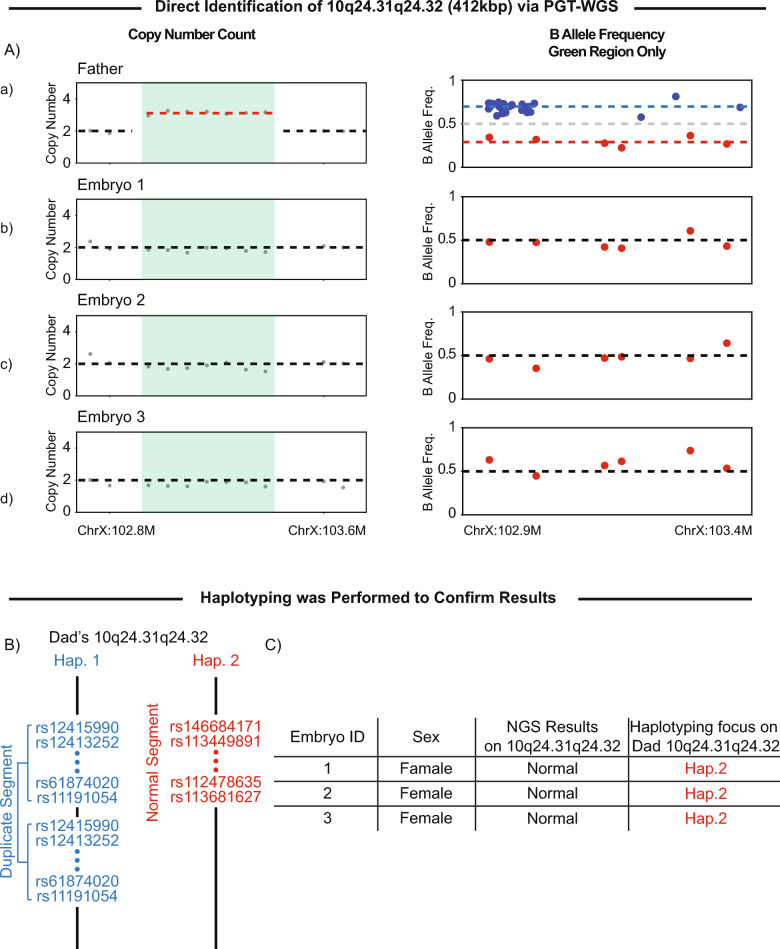
**A** Copy number analysis and B-allele frequency analysis of father and embryos, highlighting the 10q24.31q24.32 region. The B-allele frequency focuses on the 10q24.31q24.32 only. **B** Father’s haplotypes are based on the existing analysis. The identification numbers are dbSNP identification numbers of paternal single nucleotide polymorphisms seen in the allele. **C** Summary table presents the 10q24.31q24.32 results for each embryo. The haplotyping analysis confirmed the 10q24.31q24.32 findings accordingly

PGT–WGS was performed on three embryo biopsies and included screening for aneuploidy, 50 targeted microdels/dups, as well as the presence and absence of the paternal 10q24.31q24.32 duplication. A paternal sample was provided as a positive control. The duplication was identified in the paternal sample both in the copy number analysis and a splitting BAF pattern (Fig. 3A-a) but was not detected in any of the embryos. All embryos exhibited two copies of the chromosome and displayed a normal BAF pattern; therefore, they did not inherit the duplication (Fig. 3A-bcd).

To confirm these findings, additional single nucleotide polymorphisms (SNP) analysis was conducted. The results revealed that all SNPs inherited by the embryos corresponded to low BAF variants in the duplication region in the affected father (red dots), while none of the father’s high BAF variants (blue dots) were passed on (Fig. 3A). This suggests that the father carries a duplication of the chromosome segment containing the blue set of variants in one allele, along with a normal segment harboring the red set of variants in the other allele (Fig. 3B).

In addition, since DNA from both parents was available, haplotyping was performed to confirm these results further. As shown in Fig. 3C, it is confirmed that only the unaffected paternal allele was inherited by the embryos.

The PGT–WGS platform provided the couple with an additional layer of confidence as they moved forward in their journey to achieve a pregnancy.

## Discussion and conclusion

Microdeletions and microduplications are significant contributors to neurodevelopmental disorders and birth defects, yet many are not detected by traditional PGT platforms. PGT–WGS, which sequences nearly the entire genome (~3 billion sites), offers significantly more data and enables confirmatory analysis through multiple methods, including haplotyping, copy number analysis and BAF pattern for increased accuracy.

While not all patients require extensive embryo screening, PGT–WGS offers a valuable option for those seeking more comprehensive analysis or who carry a complex chromosomal condition out of the limit of detection of other platforms within a reasonable amount of time (Supplementary Table S2). To maintain clinical utility, PGT–WGS analysis is restricted to 50 common pathogenic regions.

As part of pre-test counseling, families are informed that genetic testing may reveal unexpected health-related information about themselves. This consideration applies to all forms of PGT, including PGT-A, where patterns such as segmental aneuploidy observed across multiple embryos can suggest a parental structural rearrangement, such as a translocation. In PGT–WGS, which involves more comprehensive screening, there is a greater likelihood of identifying inherited conditions. Families pursuing PGT–WGS are required to undergo thorough pretest counseling to review the potential benefits and risks before proceeding and are fully informed about the possibility of uncovering personal health findings.

With regard to technical limitations, it is important to note that—aside from fragile X syndrome—repeat expansion disorders remain difficult to detect using whole-genome sequencing (WGS) through Illumina or Complete Genomics’s platforms, a limitation inherent to short-read sequencers in general, not just to PGT–WGS. In addition, microdeletions and microduplications smaller than 300 kb are challenging to detect reliably through PGT–WGS; in such cases, traditional preimplantation genetic testing for monogenic disorders (PGT-M) with family studies and probe development remains the more appropriate approach.

At present, whole-genome sequencing (WGS) at 30× depth is inherently more expensive than conventional PGT-A, which typically utilizes sequencing at depths below 0.5×. As sequencing costs continue to decline, we anticipate that the cost of this assay will become lower over time.

Currently, patients pursuing PGT–WGS often have complex genetic conditions, many of whom have been declined by other PGT providers owing to the complexity of their cases or are seeking more comprehensive genomic screening beyond standard chromosomal counts. Therefore, we believe that this assay addresses a significant gap in current PGT offerings and has the potential to benefit a broader patient population.

In summary, PGT–WGS enables the detection of chromosomal and structural variants that may be missed by traditional PGT-A as described in case 1. It also offers the possibility of screening for challenging structural variants without requiring additional informative familial samples and/or probe development. As described in case 2, this provides an option for families who other laboratories might otherwise reject owing to technical limitations.

## Material and methods

### Sample collection and processing

Saliva samples for this study were collected from patients using AccuGene’s AccuSaliva Collection Kits (San Diego, CA, USA). DNA extraction was subsequently performed with the QIAamp DSP DNA Blood Mini Kit (Germantown, MD, USA). DNA concentration was measured using the Qubit Flex from ThermoFisher (Waltham, MA, USA). DNA integrity was assessed with the Agilent TapeStation 4150 (Santa Clara, CA, USA) using Genomic ScreenTapes. For quality assurance, DNA needed a concentration above 3.5 ng/µL and a DNA Integrity Number (DIN) greater than 5.5.

Each embryo biopsy consisted of 3–5 trophectoderm biopsy cells with a maximum of 3 µL cell buffer in a 200 µL polymerase chain reaction (PCR) tube. Whole-genome amplification was performed directly in the PCR tube as described previously [10]. DNA concentration was measured using the Qubit Flex from ThermoFisher.

For Next Generation Sequencing, between 250 and 500 ng of DNA was used to prepare sequencing libraries using the KAPA HyperPlus kit from Roche Diagnostics (Indianapolis, IN, USA) in combination with Dual Index UMI adapters from IDT (Coralville, IA, USA). Library concentration was determined using the Qubit 1X dsDNA HS assay (Waltham, MA, USA), while library sizes were assessed with the Agilent 4150 Tapestation Genomic ScreenTape (Santa Clara, CA, USA). Sequencing was performed on the Illumina MiniSeq platform for low-pass aneuploidy screening and by Psomagen (Rockville, MD, USA) on NovaSeq X Plus for 30× WGS.

Both couples in this study consented to screen all 50 microdel/dup regions included on our panel (Supplementary Table S1), including Xp22.31. These regions are well-characterized, pathogenic copy number variations (CNVs) associated with known clinical outcomes, such as neurodevelopmental delay, congenital anomalies, and seizures.

During the required pretest consultation, which is provided at no cost, patients were informed that certain findings could potentially reflect their own genetic status. The couple provided informed consent prior to testing, acknowledging this possibility. They also consented to the publication of relevant findings to contribute to the scientific community.

### Microdel/dup calling

Individual 30× depth BAM files were generated using the Gencove human WGS pipeline GRCH37 v1.0. BAM files were then loaded into NxClinical for manual review. Targeted microdel/dup regions were screened for gains and losses ≥ 400 Kb. For targeted regions presented in Supplementary Table S1, common deletions and/or duplications that fall below the limit of detection, only copy number variants larger than 400 kb would be reported.

### Haplotype analysis

A pairwise haplotype linkage analysis within the embryo cohort was performed as a confirmatory test to support sequencing results for known or suspected inherited variants. This analysis involved identifying informative single nucleotide polymorphisms (SNPs)—those present in only one parent—with a population frequency of 1–5% in the target sample, located within a 1 million base pair window surrounding the target variant in the parental gVCF, when available. X-linked SNPs were identified on male embryos in the absence of parental DNA. The variants in each pair of embryos were then compared to determine if the haplotype inheritance (sharing one, two, or both haplotypes) at a given locus was consistent with the genotypes identified through direct calls and the presumed parental origin. In cases where the gamete source of the variant was unknown, two separate analyses were conducted to confirm consistency with either maternal or paternal inheritance.

## Supplementary Information

Supplementary Material 1: Table S1. Microdel/dup list used in PGT–WGS.

Supplementary Material 2.

Supplementary Material 3: Figure S1. Case study workflow.

## Data Availability

Data will be available on request.
