# Supplementary material for: Application of microdeletion and microduplication screening in preimplantation genetic testing: a case report
Source: J Med Case Rep. 2026 Jan 22;20:99. doi: 10.1186/s13256-026-05832-3 (PMC12910960; doi:10.1186/s13256-026-05832-3)
Supplement: Supplementary file 1 — Supplementary Material 1: Table S1. Microdel/dup list used in PGT–WGS. [file 13256_2026_5832_MOESM1_ESM.pdf]

Table S1

	Hg19 Coordinates	Length (bases)	Condition
1	chr1:834,083-6,289,973	5,455,891	1p36 terminal region
2	chr1:146,577,486-147,394,506	817,021	1q21.1 recurrent region
3	chr1:243,287,730-245,318,287	2,030,558	1q43q44 terminal region
4	chr2:59,139,200-62,488,871	3,349,672	2p15p16.1
5	chr2:96,739,012-97,671,429	932,418	2p15p16.1
6	chr2:111,392,193-113,104,742	1,712,550	2q11.2 recurrent region
7	chr2:239,954,693-242,930,600	2,975,908	2q37.3 terminal region
8	chr3:195,756,054-197,344,662	1,588,609	3q29 recurrent region
9	chr4:331,568-2,010,962	1,679,395	4p16.3 terminal (Wolf-Hirschhorn syndrome) region
10	chr5:37,693-11,347,262	11,309,570	5p15 terminal (Cri du chat syndrome) region
11	chr5:175,728,979-177,047,793	1,318,815	5q35 recurrent (Sotos syndrome) region
12	chr7:5,536,848-5,799,722	262,875	7p22.1 region
13	chr7:72,744,455-74,142,510	1,398,056	7q11.23 recurrent (Williams-Beuren syndrome) region
14	chr7:75,158,048-76,063,176	905,129	7q11.23 recurrent distal region
15	chr8:8,100,064-11,766,329	3,666,266	8p23.1 recurrent region
16	chr10:81,682,843-88,739,388	7,056,546	10q22.3q23.2
17	chr11:43,894,800-46,152,450	2,257,651	11p11.2 (Potocki-Shaffer syndrome) region
18	chr11:31,803,509-32,510,988	707,480	11p13 (WAGR syndrome) region
19	chr11:67,763,646-71,236,931	3,473,286	11q13.2q13.4 recurrent region
20	chr14:21,826,900-21,861,987	35,088	14q11.2
21	chr15:22,832,519-23,090,897	258,379	15q11.2 recurrent region
22	chr15:22,832,519-28,379,874	5,547,356	15q11.2q13 recurrent (PWS/AS) region
23	chr15:31,192,889-32,445,405	1,252,517	15q13.3 recurrent region
24	chr15:32,019,621-32,445,405	425,785	15q13.3 recurrent region
25	chr15:72,963,715-75,508,312	2,544,598	15q24 recurrent region
26	chr15:83,213,988-84,714,733	1,500,746	15q25.2 recurrent proximal region
27	chr16:28,822,635-29,046,499	223,865	16p11.2 recurrent distal region
28	chr16:29,649,997-30,199,852	549,856	16p11.2 recurrent proximal region
29	chr16:21,948,445-22,430,804	482,360	16p12.2 recurrent proximal region
30	chr16:3,775,056-3,930,121	155,066	16p13.3
31	chr17:16,810,028-20,213,202	3,403,175	17p11.2 recurrent (SMS/PLS) region
32	chr17:14,097,915-15,422,952	1,325,038	17p12 recurrent (HNPP/CMT1A) region
33	chr17:1,247,833-2,588,909	1,341,077	17p13.3 (Miller-Dieker syndrome) region
34	chr17:29,097,069-30,264,027	1,166,959	17q11.2 recurrent region
35	chr17:34,815,072-36,192,489	1,377,418	17q12 recurrent (RCAD) region
36	chr17:43,705,166-44,164,880	459,715	17q21.3 (Koolen-de Vries syndrome) Region
37	chr17:58,113,002-60,275,809	2,162,808	17q23.1q23.2
38	chr22:18,912,231-20,287,208	1,374,978	22q11.2 recurrent (DGS/VCFS) region
39	chr22:20,731,986-21,465,672	733,687	22q11.2 recurrent central region
40	chr22:21,917,117-23,649,111	1,731,995	22q11.2 recurrent distal region
41	chr22:23,831,202-24,632,821	801,620	22q11.2 recurrent (SMARCB1) region
42	chrX:624,344-659,411	35,068	Xp22.33 (SHOX) region
43	chrX:31,137,345-33,229,673	2,092,329	Xp21.2p21.1 (DMD) region
44	chrX:53,363,456-53,793,054	429,599	Xp11.22
45	chrX:48,306,152-52,103,258	3,797,107	Xp11.22p11.23 recurrent region
46	chrX:43,514,154-43,741,720	227,567	Xp11.23
47	chrX:30,195,000-30,355,000	160,001	Xp21.2
48	chrX:6,455,812-8,124,954	1,669,143	Xp22.31
49	chrX:154,118,603-154,564,401	445,799	Xq28
50	chrX:153,273,980-153,375,749	101,770	Xq28
