# Supplementary material for: Application of microdeletion and microduplication screening in preimplantation genetic testing: a case report
Source: J Med Case Rep. 2026 Jan 22;20:99. doi: 10.1186/s13256-026-05832-3 (PMC12910960; doi:10.1186/s13256-026-05832-3)
Supplement: Supplementary file 2 — Supplementary Material 2. [file 13256_2026_5832_MOESM2_ESM.docx]

**Table S2.** Timeline of the reported cases.

|  | Pre-Test Consultation | Sample Received | Report  Delivered | Post-Test Consultation |
| --- | --- | --- | --- | --- |
| Case 1 | 02/27/2024 | 04/03/2024 | 04/25/2024 | 04/29/2024 |
| Case 2 | 01/30/2024 | 05/22/2024 | 06/19/2024 | 06/21/2024 |
