# Supplementary figures and images for: Application of microdeletion and microduplication screening in preimplantation genetic testing: a case report
Source: J Med Case Rep. 2026 Jan 22;20:99. doi: 10.1186/s13256-026-05832-3 (PMC12910960; doi:10.1186/s13256-026-05832-3)

Figure S1

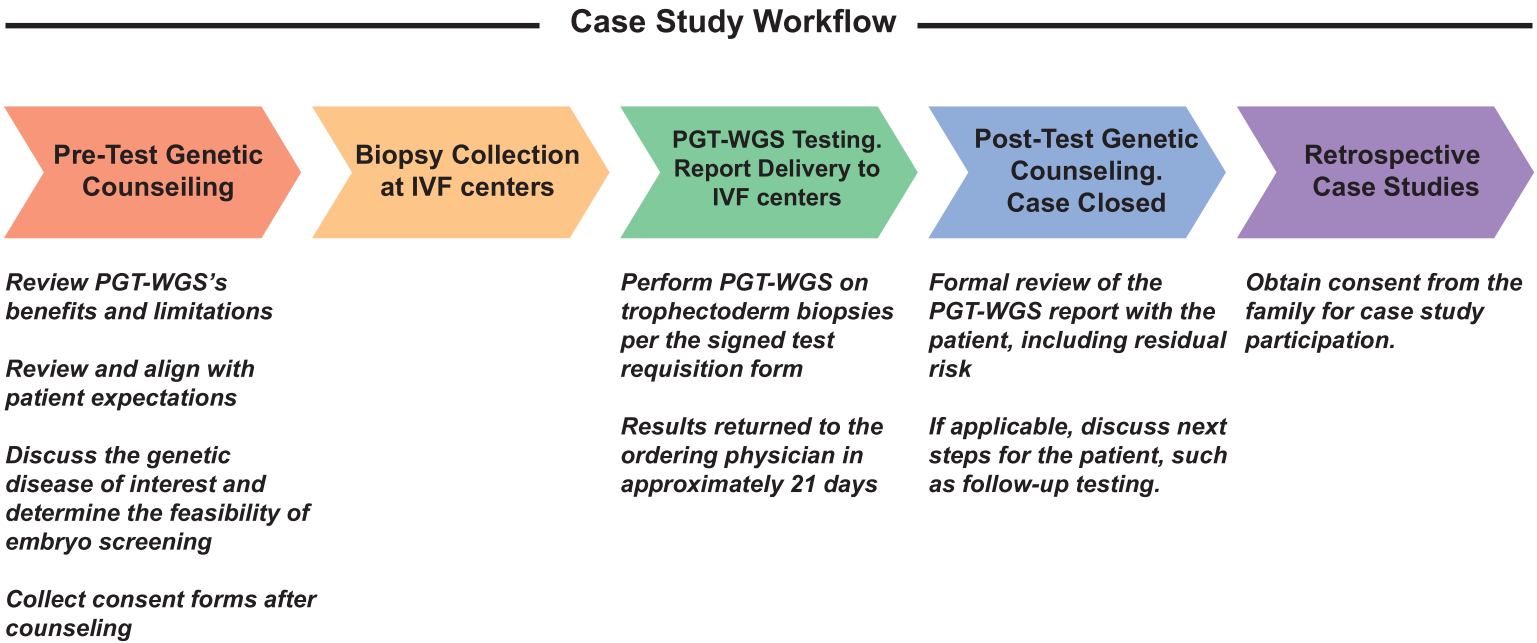

Supplement: Supplementary file 3 — Supplementary Material 3: Figure S1. Case study workflow. [file 13256_2026_5832_MOESM3_ESM.pdf]
